# Longitudinal Trends of Hospitalizations for Giant Cell Arteritis: A 21-Year Longitudinal National Population-Based Study

**DOI:** 10.7759/cureus.35038

**Published:** 2023-02-15

**Authors:** Christopher Hino, Ehizogie Edigin, Osaigbokan Aihie, Jesse Odion, Precious Eseaton, Victory Okpujie, Precious Onobraigho, Eugene Omoike, Augustine Manadan, Mehrnaz Hojjati

**Affiliations:** 1 Department of Internal Medicine, Loma Linda University School of Medicine, Loma Linda, USA; 2 School of Medicine, University of Missouri School of Medicine, Columbia, USA; 3 Department of Internal Medicine, University of Benin College of Medicine, Benin City, NGA; 4 Department of Rheumatology, Rush University Medical Center, Chicago, USA

**Keywords:** mortality, population based study, longitudinal trends, vasculitis, giant cell arteritis (gca), giant cell arteritis

## Abstract

Background

Long-term longitudinal studies on giant cell arteritis (GCA) hospitalizations are limited. Here we aim to fill gaps in knowledge by analyzing longitudinal trends of GCA hospitalizations over the last two decades in the United States (U.S.).

Materials and methods

We performed a 21-year longitudinal trend analysis of GCA hospitalizations using data obtained from the National Inpatient Sample (NIS) database between 1998 and 2018. Using the NIS database, we searched for hospitalizations for patients aged ≥ 50 years with a principal diagnosis of GCA using ICD billing codes. The principal diagnosis was the main reason for hospitalization. We used all hospitalizations in patients without GCA aged ≥50 years as the control population. Multivariable logistic and linear regression analysis was utilized to calculate the adjusted p-trend for outcomes of interest.

Results

The incidence of GCA hospitalization remained stable at about one per 100,000 U.S. persons throughout the study period. There was no statistically significant change in the inpatient mortality for the GCA group during the study period (adjusted p-trend=0.111). In comparison, inpatient mortality reduced from 4.4% to 3.1% from 1998 to 2018 (adjusted p-trend <0.0001) in the control group. The proportion of whites reduced, while the proportion of racial minorities increased over time in both the GCA and control groups.

Conclusion

The non-GCA control population saw significant reductions in mortality over time, but unfortunately, the GCA group did not see such improvements. More research into additional treatment modalities for inpatient GCA management may help improve mortality.

## Introduction

Giant cell arteritis (GCA) is a granulomatous large vessel vasculitis that presents almost exclusively in patients older than 50 years of age. The disease typically manifests as new-onset headaches, jaw claudication, myalgias, fatigue, fevers, weight loss, and vision loss [1]. For many years, glucocorticoids and methotrexate were the only treatment options for GCA. Additionally, temporal artery biopsy was the only reliable method to diagnose GCA. However, in recent years, there have been significant advancements in GCA management including diagnostic ultrasound and interleukin-6 blockade with tocilizumab [2,3].

Despite these advances, it remains unclear if the outcomes for GCA hospitalizations have improved. Information detailing the longitudinal trends of GCA hospitalizations has been limited in part due to its relatively low prevalence [4,5]. To date, there has been no comprehensive nationwide study to evaluate trends in the epidemiology, incidence, and outcomes of GCA hospitalizations in the United States (U.S.). In this study, we provide a detailed and comprehensive evaluation of the longitudinal trends of GCA hospitalizations in the U.S. between 1998 and 2018 using a national U.S. population-based database.

The work presented here was previously presented at the American College of Rheumatology Convergence 2022 conference [6].

## Materials and methods

Database description

The National (Nationwide) Inpatient Sample (NIS) was created by the Healthcare Cost and Utilization Project (HCUP) from the Agency for Healthcare Research and Quality and is considered to be the largest publicly available all-payer hospitalization database in the U.S. [7]. The NIS is a hospital discharge database that encompasses a representative, stratified sample of nearly 20% of US community hospitals. Patient information in the NIS database is de-identified but contains patient and hospital demographic, as well as information pertaining to billing, diagnostics, and procedures. The NIS data additionally includes weights to produce national or regional estimates.

We acquired data on patient hospitalizations from the NIS database between 1998 and 2018. The methods described are similar to a previously published NIS longitudinal study [8]. In order to assess longitudinal hospitalization trends between 1998-2018, we collected data in five-year intervals during the study period, including years 1998, 2003, 2008, 2013, and 2018. Due to a redesign in the NIS data collection method in 2012 to allow for better generalizability, revised trend weights were used for data obtained before 2012 per recommendations from the HCUP methods series [9]. Per recommendations from the HCUP methods series, revised trend weights were thus utilized for NIS data acquired before 2012 to make estimates comparable across the years in our study time period [9].

The International Classifications of Diseases (ICD) diagnosis code in the NIS database was utilized to identify the principal diagnosis or main reason for hospitalization. Prior to the last quarter of 2015, the NIS utilized ICD 9th Edition (ICD-9) for diagnosis identification, while ICD 10th Edition (ICD-10) coding was utilized thereafter. As a result, ICD-9 codes were utilized in our NIS data collection for the years 1998, 2003, 2008, and 2013, while ICD-10 codes were used for the 2018 NIS data.

Ethical consideration

Patient data obtained from the NIS database is de-identified and publicly available, thus institutional review board (IRB) review and approval were not needed to conduct the following research.

Study population

We evaluated patients who had been hospitalized aged≥ 50 years with a principal diagnosis of GCA using ICD-9 code “446.5” or ICD-10 codes “M31.5” or “M31.6.” Since the “principal” diagnosis was the main reason for hospitalization, these hospitalizations can reasonably be attributed to either a new GCA diagnosis or a GCA flare. For our study’s control population, we evaluated patients who had been hospitalized aged ≥50 years without any diagnosis of GCA.

Outcomes

The primary outcome was longitudinal trends in inpatient mortality for GCA. Secondary outcomes included longitudinal trends in the incidence of GCA hospitalizations, Charlson comorbidity index (CCI) score, hospital length of stay (LOS), total hospital charges, as well as patient demographics (age, gender, and race/ethnicity) over the study period. The annual incidence of GCA hospitalizations was calculated by dividing the number of GCA hospitalizations reported in the NIS database by the estimated U.S. population obtained from the U.S Census Bureau Website on July 1 of the corresponding year [10]. Patient race was categorized between Whites, Blacks, Hispanics, and Asian/Pacific Islanders according to the reported HCUP database element description [7]. All hospital charges were adjusted for inflation by using the medical expenditure panel survey-based factor for hospital care and presented in United States Dollars (USD) [11].

Statistical analysis

STATA version 16 was utilized for the statistical analysis. Multivariable logistic and linear regression analyses were used to calculate adjusted p-trends for categorical and continuous outcomes, respectively. Categorical variables include race, sex, CCI score category (0-2 and ≥3), and inpatient mortality, while continuous variables include incidence, age, LOS, and total hospital charges. To adjust for changes in demographics and the comorbidity burden of the populations over time, demographic variables (age, sex, race) and CCI score were included in the regression models. All p-values were two-sided, and 0.05 was the threshold for statistical significance.

## Results

The incidence of GCA hospitalization remained stable at about one per 100,000 U.S persons throughout the study period (Table 1). There was no statistically significant change in the inpatient mortality for the GCA group during the study period (adjusted p-trend=0.111).

**Table 1:** Characteristics and longitudinal trends of GCA hospitalizations from 1998 to 2018 Abbreviations: CCI: Charlson comorbidity index, GCA: Giant cell arteritis, I: Incidence per 100,000 persons, LOS=Length of hospital stay; PI= Pacific Islander, USD: United States Dollars; p<0.005 was statistically significant. * We included hospitalizations for patients aged ≥50 years with a principal diagnosis of GCA ** The incidence of GCA hospitalizations for GCA was calculated by dividing the number of GCA hospitalizations in the NIS by the population estimate of the U.S on July 1 of the corresponding year. The population estimates were obtained from the U.S Census Bureau Website *** All hospital charges were adjusted for inflation by using the medical expenditure panel survey-based factor for hospital care and were presented in 2018 United States dollars (USD).

Variables	1998	2003	2008	2013	2018	Adjusted p trend
GCA hospitalizations*						
n	2,935	2,911	2,770	2,275	2,695	0.781
I, 100,000**	1.1	1	0.9	0.7	0.8	
Female, %	77.8	76.2	69.6	70.3	67.5	0.041
Mean Age, years	74.9	75.6	75.2	74	73.7	0.019
Race						
White, %	80.8	77.1	74.1	71.4	69.2	<0.0001
Black, %	10.5	12.5	11.9	14.5	17	0.024
Hispanic, %	4.7	7	8	8.5	7.4	0.049
Asian or PI, %	1.2	0.8	1.6	3.5	2.8	<0.0001
CCI score						<0.0001
0-2, %	93.5	89.2	83.9	74.1	65.1	
≥3, %	6.5	10.8	16.1	25.9	34.9	
Deceased						0.111
n	31	14	9	15	10	
%	1	0.5	0.3	0.7	0.4	
Mean LOS, days	5.8	5.1	4.8	4.4	4.3	<0.0001
Mean total Charges, USD ***	20,564	26,793	32,709	38,992	48,855	<0.0001

**Figure 1:**
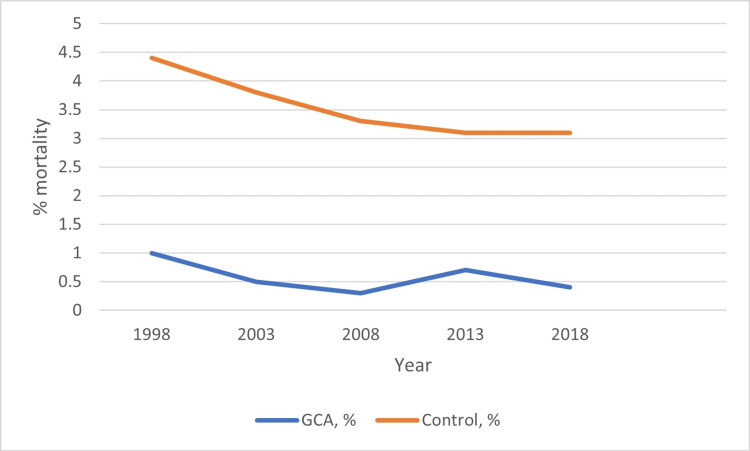
Inpatient mortality for GCA and non-GCA hospitalizations aged ≥ 50 years from 1998 to 2018 GCA: Giant cell arteritis, Control: Non-GCA hospitalizations

In comparison, the inpatient mortality for the control group improved from 4.4% to 3.1% from 1998 to 2018 (adjusted p-trend < 0.0001) (Table 2, Figure 1). The proportion of whites reduced, while the proportion of racial minorities increased over time in both the GCA and control groups. There was a statistically significant reduction in mean LOS and an increase in total hospital charges and CCI score over time in both groups (Tables 1, 2).

**Table 2:** Characteristics and longitudinal trends of non-GCA hospitalizations from 1998 to 2018 Abbreviations: CCI: Charlson comorbidity index, GCA: Giant cell arteritis, I: Incidence per 100,000 persons, LOS=Length of hospital stay; PI= Pacific Islander, USD: United States Dollars; p<0.005 was statistically significant. * We included all hospitalizations in those aged ≥50 years without any diagnosis of GCA as the control population. ** All hospital charges were adjusted for inflation by using the medical expenditure panel survey-based factor for hospital care and were presented in 2018 United States dollars (USD).

Variables	1998	2003	2008	2013	2018		Adjusted p trend
Non-GCA hospitalizations*							
n, million	17.3	19	20.4	19.6		20.3	
Female %	55.4	55.5	54.5	53.1		51.4	<0.0001
Mean Age, years	71.5	71	70.7	70		70.2	<0.0001
Race, %							
White	81.4	75.8	76.9	74.8		73	<0.0001
Black	10.2	11.4	10.9	12.7		13.1	0.015
Hispanic	5.7	8.5	6.6	7.6		8.6	0.002
Asian or P.I.	1.3	2.1	2.1	2.1		2.3	<0.0001
CCI score							<0.0001
0-2, %	81.3	81.1	73.2	67.2		57.7	
≥3, %	18.9	18.9	26.9	32.8		42.3	
Deceased							<0.0001
n	753,915	722,610	684,219	598,540		622,045	
%	4.4	3.8	3.3	3.1		3.1	
Mean LOS, days	5.8	5.5	5.3	5.1		5.2	<0.0001
Mean total Charges, USD **	26,587	37,245	44,598	52,500		64058	<0.0001

## Discussion

Over the last several decades, the treatment landscape for rheumatologic disease has changed dramatically due to improved diagnostic tools and the introduction of targeted biological therapies. As a result, the U.S. has witnessed a steady decline in rheumatologic disease severity and related mortality [12-14]. To elucidate whether similar changes have occurred in GCA, we studied longitudinal trends for GCA hospitalizations in the U.S. over the last two decades using the NIS database.

Our results demonstrate that the overall incidence of GCA hospitalizations remained relatively stable over the last 2 decades. Consistent with previous studies, patients hospitalized for GCA were predominantly white females in their 70s. However, we found the mean age significantly decreased over time for both the GCA and control populations. More interestingly we found the proportion of whites decreased over time while ethnic minorities such as Black, Hispanic, and Asian or Pacific Islander patients increased in both the GCA and control populations. The racial trends seen in the GCA group were similar to the general U.S. population which also saw an increase in the proportion of ethnic minorities over time [15]. Our findings may also be due to the increasing recognition of GCA in ethnic minorities. The previous underrecognition of GCA in ethnic minorities may be due to the fact that many previous studies were predominantly in white populations in Europe and North America [16-24].

Over the 21-year period, the mean LOS decreased but the mean total hospital charges and CCI score increased in both the GCA and control population. These results are consistent with previously reported trends in the U.S. showing an overall rise in healthcare costs, a shortening LOS, and an increasing comorbidity burden of the U.S. population over time [25]. While inpatient mortality improved in the control group between 1998-2018, we found no such improvements in the GCA group. These findings could be due to low GCA inpatient mortality which ranged from 0.3-1% in our study. In comparison, the control population had much higher inpatient mortality ranging from 5.1-5.8%. With advances in diagnostic tools and the introduction of tocilizumab, we were surprised to find that GCA inpatient mortality did not significantly improve over time. However, we acknowledge that the present study only evaluated data through the end of 2018, which is less than 2 years after FDA approval of tocilizumab for GCA. It may therefore be too early to appreciate the mortality benefit of tocilizumab in GCA. Also, tocilizumab is mostly started as a corticosteroid-sparing agent in the outpatient setting after hospital discharge. Hence corticosteroids are still the mainstay of in-hospital GCA treatment. Further research into additional treatment regimens for inpatient GCA management is needed to further reduce inpatient mortality.

The GiACTA trial demonstrated the superiority of the combination of tocilizumab and corticosteroids versus placebo and corticosteroids in terms of sustained corticosteroid-free remission but did not report changes in disease-related mortality [3]. Given that GCA mortality is primarily due to glucocorticoid-related complications (e.g., infections, cardiovascular disease, endocrinopathies), it is still reasonable to hypothesize that tocilizumab may reduce GCA mortality by decreased glucocorticoid use over time [26]. In addition, not all patients with GCA receive tocilizumab due to contraindications, physician judgment or patient preference. Hence it may take a longer time before we can see any significant change in the mortality and morbidity of patients with GCA. Further long-term clinical studies that assess trends in morbidity of GCA patients using objective measurements are needed to evaluate the effect of tocilizumab use among patients with GCA in more recent times.

There are several strengths to our study. Firstly, the use of a U.S nationally representative inpatient database enabled us to evaluate the epidemiological characteristics and longitudinal trends of GCA hospitalizations of the U.S. population over a 21-year period. Secondly, the large sample size provided by the NIS database provided much-needed statical power to evaluate a relatively rare disease. Our study is not without limitations. NIS is a claims database based on ICD codes, hence there is a possibility of some errors due to coding. The NIS reports data on hospitalizations, rather than on individual patients. Hence patients hospitalized on multiple occasions will be counted multiple times [8]. The NIS does not contain data on GCA disease severity, treatment regimens, medication compliance, laboratory information such as inflammatory markers, imaging results, temporal artery biopsy results, and clinical outcomes after discharge.

## Conclusions

In the U.S., the incidence of GCA hospitalizations has remained stable over the last two decades. The inpatient mortality of GCA hospitalizations has not changed significantly over time in contrast to the control population which saw significant improvements in mortality. Other trends were similar between both groups. Although tocilizumab has been approved for the treatment of GCA, corticosteroids remain the mainstay of acute GCA management. More research into additional treatment modalities for acute GCA may help further reduce inpatient mortality.
